# A comparison of prognostic significance of strong ion gap (SIG) with other acid-base markers in the critically ill: a cohort study

**DOI:** 10.1186/s40560-016-0166-z

**Published:** 2016-06-29

**Authors:** Kwok M. Ho, Norris S. H. Lan, Teresa A. Williams, Yusra Harahsheh, Andrew R. Chapman, Geoffrey J. Dobb, Sheldon Magder

**Affiliations:** Department of Intensive Care Medicine, Royal Perth Hospital, Wellington Street, Perth, WA 6000 Australia; School of Population Health, University of Western Australia, Perth, Australia; School of Veterinary and Life Sciences, Murdoch University, Perth, Australia; School of Medicine and Pharmacology, University of Western Australia, Perth, Australia; School of Nursing, Midwifery and Paramedicine, Curtin University, Perth, Australia; Critical Care Division, Department of Medicine and Physiology, Royal Victoria Hospital, McGill University Health Centre and McGill University, Montréal, Canada

**Keywords:** Acidosis, Anion gap, Alkalosis, Outcomes, Strong ion difference

## Abstract

**Background:**

This cohort study compared the prognostic significance of strong ion gap (SIG) with other acid-base markers in the critically ill.

**Methods:**

The relationships between SIG, lactate, anion gap (AG), anion gap albumin-corrected (AG-corrected), base excess or strong ion difference-effective (SIDe), all obtained within the first hour of intensive care unit (ICU) admission, and the hospital mortality of 6878 patients were analysed. The prognostic significance of each acid-base marker, both alone and in combination with the Admission Mortality Prediction Model (MPM_0_ III) predicted mortality, were assessed by the area under the receiver operating characteristic curve (AUROC).

**Results:**

Of the 6878 patients included in the study, 924 patients (13.4 %) died after ICU admission. Except for plasma chloride concentrations, all acid-base markers were significantly different between the survivors and non-survivors. SIG (with lactate: AUROC 0.631, confidence interval [CI] 0.611–0.652; without lactate: AUROC 0.521, 95 % CI 0.500–0.542) only had a modest ability to predict hospital mortality, and this was no better than using lactate concentration alone (AUROC 0.701, 95 % 0.682–0.721). Adding AG-corrected or SIG to a combination of lactate and MPM_0_ III predicted risks also did not substantially improve the latter’s ability to differentiate between survivors and non-survivors. Arterial lactate concentrations explained about 11 % of the variability in the observed mortality, and it was more important than SIG (0.6 %) and SIDe (0.9 %) in predicting hospital mortality after adjusting for MPM_0_ III predicted risks. Lactate remained as the strongest predictor for mortality in a sensitivity multivariate analysis, allowing for non-linearity of all acid-base markers.

**Conclusions:**

The prognostic significance of SIG was modest and inferior to arterial lactate concentration for the critically ill. Lactate concentration should always be considered regardless whether physiological, base excess or physical-chemical approach is used to interpret acid-base disturbances in critically ill patients.

## Background

Acid-base disturbances due to either the underlying pathological process or intensive care therapy are common in critically ill patients. Broadly speaking, there are three approaches to assess acid-base disturbances, including the physiological approach, the base excess (BE) approach and the physical-chemical approach [1]. The physiological approach uses the Henderson-Hasselbalch equation, and arterial pH is assumed to be solely determined by the balance between arterial carbon dioxide tension (respiratory component) and plasma bicarbonate concentration (metabolic component). The BE approach has some similarities to the physiological approach, but it uses the BE instead of bicarbonate to define the metabolic component of acid-base disturbances. BE is estimated by how much acid or base is needed to adjust the pH back to 7.40 while correcting the arterial carbon dioxide tension to 40 mmHg. Further refinement of these approaches includes anion gap (AG) with and without correcting for hypoalbuminaemia (AG-corrected) to define whether excessive anions other than chloride (Cl^−^) and bicarbonate are present.

The third approach to quantify acid-base disturbances is the physical-chemical approach, also called Stewart’s acid-base approach. In this approach, the three main determinants of acid-base status are (i) total carbon dioxide content in vitro (representing carbon dioxide in both dissolved and undissolved forms) or partial pressure of carbon dioxide in vivo, (ii) the weak non-volatile acids (mainly albumin and phosphate) and (iii) the strong ion difference. Strong ion difference (SID-apparent or SIDa) is the difference between the amount of fully dissociated cations (Na^+^, K^+^, Ca^2+^, Mg^2+^) and anions (Cl^−^). The physical-chemical approach to acid-base is more comprehensive than the other two approaches and can identify subtle or combined acid-base disturbances that are not apparent using the physiological or BE approach alone [2, 3]. Furthermore, recent evidence suggested that abnormalities in SIDa or strong ion gap (SIG) are associated with severity of inflammation, suggesting that abnormal Stewart’s acid-base status may have pathogenic consequences and hence prognostic significance [4]. Stewart’s approach to acid-base disturbances is, however, more complicated than the physiological and BE approaches, and unless such data are automatically generated from the laboratory, a smartphone application may be needed to facilitate interpretation [1].

We hypothesised that SIG may be more important than the other acid-base markers, and Stewart’s approach to acid-base disturbances may be superior to other markers of acid-base status in predicting mortality of critically ill patients [5, 6]. If this is the case, it would be essential for SIG to be determined and monitored regularly in critically ill patients. In this cohort study, we compared the prognostic significance of SIG with other commonly used acid-base markers and determined whether SIG is better than other acid-base markers, either by itself or when combined with a validated prognostic model, in predicting hospital mortality of the critically ill.

## Methods

In this study, the physiological and biochemical data of the patients on admission to the ICU at the Royal Perth Hospital Intensive Care Unit (ICU) between 1 January 2008 and 31 December 2013 were analysed. Royal Perth Hospital is a 450-bed university teaching hospital, and the 20-bed ICU is a tertiary ICU, staffed by fully trained intensivists, admitting critically ill adult patients of all specialties with the exception of liver transplantation. The dataset contained all the components of the Simplified Acute Physiology Score (SAPS) III [7], Acute Physiology and Chronic Health Evaluation (APACHE) II score [8] and Admission Mortality Prediction Model (MPM_0_ III) [9], as well as biochemical data required to estimate AG, SIDa and SIG in the blood tests obtained within the first hour of ICU admission [10]. The data on SIG and SIDa were not automatically generated by the laboratory, and none of the clinicians in the study centre used Stewart’s approach to diagnose and manage acid-base disturbances.

The study data were checked for transcription errors and completeness by a designated trained clerical staff member, using data from the computerised laboratory database and going through the ICU vital signs flow chart again before the data were transferred to the computer. A single data custodian was responsible for ensuring data quality. The data were reviewed for internal consistency, and there were no patients lost to follow-up or with missing hospital mortality data. This study utilised only clinical data that were de-identified and all ICU readmissions during the same hospitalisation were excluded, was registered as a clinical audit with the Clinical Safety and Quality Unit (150521-02) and was exempt from review by the Royal Perth Hospital Ethics Committee.

In this study, we compared the prognostic significance of SIG with other commonly used acid-base markers, including pH, carbon dioxide tension, actual (calculated) bicarbonate, Cl^−^, lactate, AG, AG-corrected, actual (calculated) BE, SID-effective and SIG (with [5] and without including lactate as part of SIG [3]) (Radiometer®, Copenhagen, Denmark), using arterial blood specimens of the study patients all obtained within the first hour of ICU admission. In addition, other ions that were unmeasured, also called the ‘other’ or ‘other unmeasured ions’ [1], were also estimated by subtracting the water, Cl^−^ and protein effect from the BE. The methods to estimate and calculate AG, AG-corrected, SIDa (with and without including lactate in the calculations), SID-effective (SIDe), SIG (with and without including lactate in the calculations) and ‘other unmeasured ions’ (or BE gap) are described in Appendix 1.

### Statistical analysis

After confirming that the acid-base markers did not have an extreme U-shape relationship with the observed risks of death (Appendix 2) which may compromise the accuracy of the area under the receiver operating characteristic curve (AUROC) analysis [11], AUROC was used to compare the discrimination ability of different acid-base markers. The difference in AUROC between different acid-base markers derived from the same patients was calculated by the *z* statistic as described by Hanley and McNeil [12]. We then assessed whether each of these acid-base markers would improve the ability of the MPM_0_ III model to predict hospital mortality of the critically ill patients by combining each of these markers with the predicted risks of MPM_0_ III [13], also by AUROC. In addition, we also assessed whether SIG (with lactate included as part of SIG) or SIDe was superior to arterial lactate concentration in explaining the variability in the observed hospital mortality, based on each predictor’s chi square contribution in a multivariate logistic regression [14], while adjusting for the MPM_0_ III predicted risks. The MPM_0_ III prognostic model was primarily chosen as the preferred risk adjustment model in this study because it does not utilise any laboratory markers of acid-base status, and hence, no one acid-base marker was favoured in terms of its prognostic significance due to multicollinearity with the risk adjustment prognostic model in the AUROC and logistic regression analyses.

Finally, four separate sensitivity analyses were performed; one analysis was on patients with known cirrhosis to assess whether SIG, SIDe or bilirubin concentration was better than arterial lactate in predicting mortality as this specific subgroup of patients was known to have substantial accumulation of unmeasured anions [15, 16]. The second sensitivity analysis was conducted by replacing the MPM_0_ III predicted risks with the SAPS III predicted risks to assess whether SIG or SIDe would be better than lactate concentrations when a different risk adjustment tool was used. The third sensitivity analysis was to assess whether the AUROC of the predictors that had some degree of U-shape relationship to mortality (pH, chloride, bicarbonate and arterial carbon dioxide tension) (Appendix 2) would change substantially after centring these predictors [11]. In the last sensitivity analysis, we analysed all acid-base markers in a multivariate analysis, allowing non-linearity by a 3-knot restricted cubic spline function for all acid-base markers [14, 17].

In this study, a *p* value < 0.05 was taken as significant and all statistical analyses were performed by SPSS for Windows (version 22.0, IBM, USA), MedCalc for Windows (version 12.5, Ostend, Belgium) or S-PLUS (version 8.0, 2007; Insightful Corp., Seattle, WA, USA).

### Availability of data and materials

The SPSS dataset supporting the findings of this study will be provided if the readers contact the corresponding author.

## Results

**Fig. 1:**
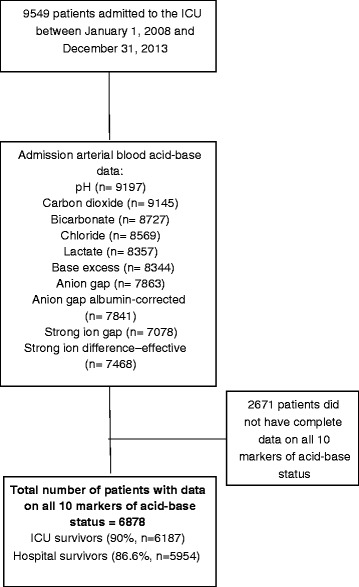
Flow chart showing inclusion and exclusion of patients in this study

Of the 6878 patients included in the study (Fig. 1), 924 patients (13.4 %) died during the same hospitalisation after ICU admission. The patients who died were older, with more comorbidities and a higher acuity of acute illness (Table 1). Except for plasma chloride concentrations, all acid-base markers on admission to the ICU were significantly different between hospital survivors and non-survivors (Table 2).

**Table 1:** Characteristics of the study cohort

Variable	Total cohort (*n* = 6878)	Survivors (*n* = 5954)	Non-survivors (*n* = 924, 13.4 %)	*p* value^a^
Age, years (IQR)	54.4 (38–69)	53.2 (36–67)	62.9 (48–75)	0.001
Male, no. (%)	4504 (66)	3930 (66)	574 (62)	0.023
ICU admission source, no. (%)				0.001
- Operating theatre	3166 (46)	2919 (49)	247 (27)	
- Emergency department	1813 (26)	1508 (25)	305 (33)	
- Ward	574 (8)	422 (7)	152 (16)	
- CCU/HDU	286 (4)	213 (4)	73 (8)	
- Other hospital	930 (14)	806 (14)	124 (13)	
- Other hospital ICU	55 (1)	42 (1)	13 (1.4)	
Elective surgery, no. (%)	1881 (27)	1798 (30)	83 (9)	0.001
Ward stay before ICU, days (IQR)	5 (2–10)	5 (2–10)	4 (2–13)	0.958
Mechanical ventilation on adm (%)	5412 (79)	4677 (79)	735 (80)	0.383
Acute renal failure on adm, no. (%)^b^	392 (6)	185 (3)	207 (22)	0.001
Worst 24-h APACHE II score (IQR)	17.0 (12–22)	16 (12–21)	27 (21–32)	0.001
SAPS III score (IQR)	43 (34–54)	41 (33–50)	60 (51–68)	0.001
SAPS III predicted risk, % (IQR)	7.9 (3–22)	6.3 (2–16)	32.8 (17–49)	0.001
MPM_0_ III predicted risk, % (IQR)	15.7 (8–31)	13.9 (7–26)	41.5 (22–68)	0.001
ICU stay, days (IQR)	3 (2–6)	3 (2–6)	4 (2–7)	0.001
Hospital stay, days (IQR)	13 (7–25)	14 (8–26)	6 (3–17)	0.001
Chronic medical conditions (%)^b^				
- Respiratory	314 (5)	264 (4)	50 (5)	0.203
- Cardiovascular	679 (10)	579 (10)	100 (11)	0.313
- Liver	167 (2)	127 (2)	40 (4)	0.001
- Renal	323 (5)	244 (4)	79 (9)	0.001
- Immune disease	69 (1)	51 (0.9)	18 (2)	0.004
- Immune treatment	252 (4)	185 (3)	67 (7)	0.001
- Metastatic cancer	93 (1)	71 (1)	27 (2)	0.008
- Lymphoma	39 (0.6)	25 (0.4)	14 (2)	0.001
- Leukaemia/myeloma	83 (1)	53 (0.9)	30 (3)	0.001
- AIDS	7 (0.1)	3 (0.05)	4 (0.4)	0.008
Major admission diagnoses, no. (%)				
Cardiac or respiratory arrest	345 (5)	182 (3)	163 (18)	0.001
Pneumonia	265 (4)	23 (4)	42 (5)	0.233
Septic shock	36 (6)	324 (5)	112 (12)	0.001
Multiple trauma	491 (7)	455 (8)	36 (4)	0.001
Isolated head trauma	620 (9)	526 (9)	94 (10)	0.195
Intracranial haemorrhage	235 (3)	154 (3)	81 (9)	0.001
Drug overdoses	449 (7)	441 (7)	8 (0.9)	0.001
Congestive heart failure, ischaemic heart disease or cardiogenic shock	179 (3)	129 (2)	50 (5)	0.001
Peripheral vascular disease or aortic aneurysm	205 (3)	184 (3)	21 (2)	0.211
GI obstruction or perforation	161 (2)	134 (2)	27 (3)	0.200
Aspiration pneumonia	76 (1)	68 (1)	8 (0.9)	0.611
Obstructive airway disease	136 (2)	127 (2)	9 (1)	0.015
Heart valve surgery	516 (8)	503 (8)	13 (1)	0.001
Coronary artery bypass graft surgery	982 (14)	958 (16)	24 (3)	0.001
Acute lung injury or ARDS	27 (0.4)	22 (0.4)	5 (0.5)	0.398
Gastrointestinal bleeding	125 (2)	106 (2)	19 (2)	0.511
Pulmonary embolism	22 (0.3)	16 (0.3)	6 (0.6)	0.106

All values are median and interquartile range (IQR) in parenthesis unless stated otherwise

*Adm* admission, *GI* gastrointestinal, *APACHE* Acute Physiology and Chronic Health Evaluation, *ARDS* acute respiratory distress syndrome, *CCU* coronary care unit, *HDU* high dependency unit, *ICU* intensive care unit, *MPM*_*0*_ *III* Mortality Prediction Model on admission, *SAPS* Simplified Acute Physiology Score

^a^ *p* values generated by either Mann-Whitney or chi square test

^b^According to the definitions by the APACHE model

**Table 2:** Differences in different markers of acid-base disorders at ICU admission between survivors and non-survivors (*n* = 6878)

Acid-base markers	Survivors (*n* = 5954)	Non-survivors (*n* = 924)	*p* value^a^
1. Arterial pH	7.35 (7.29–7.39)	7.28 (7.17–7.37)	0.001
2. Arterial CO_2_ tension, mmHg	40 (35–45)	40 (34–48.8)	0.022
3. Actual bicarbonate conc., mmol/L	21 (19–23)	18 (14–21.8)	0.001
4. Chloride conc., mmol/L	110 (107–113)	109 (105–114)	0.891
5. Lactate conc., mmol/L	1.5 (1.0–2.4)	2.7 (1.4–5.6)	0.001
6. Actual base excess, mmol/L	−3 (−6 to −1)	−7 (−12 to −3)	0.001
7. Anion gap, mmol/L	12.5 (10.1–15.0)	15.0 (11.9–19.5)	0.001
8. Anion gap albumin-corrected, mmol/L	15.5 (12.8–18.5)	18.7 (14.8–23.6)	0.001
9. SIG with lactate, mmol/L	4.2 (1.5–7.1)	6.5 (3.0–10.8)	0.001
10. SIG without lactate, mmol/L	2.2 (−0.3 to 5.0)	2.5 (−0.4 to 5.8)	0.028
11. SID-effective, mmol/L	33.5 (30.5–36.2)	30.7 (26.7–34.7)	0.001
12. Other unmeasured ions, mmol/L	2.1 (−1.0 to 4.9)	−1.5 (−7.3 to 2.5)	0.001

All data are median values with the interquartile range reported in parenthesis

*CO*_*2*_ carbon dioxide, *SID* strong ion difference, *SIG* strong ion gap

^a^ *p* values generated by Mann-Whitney test

SIG (with lactate: AUROC 0.631, confidence interval [CI] 0.611–0.652; without lactate: AUROC 0.521, 95 % CI 0.500–0.542) only had a modest ability to predict hospital mortality, and this was no better than using lactate concentration alone (AUROC 0.701, 95 % 0.682–0.721). Arterial lactate concentration, both by itself and in combination with the MPM_0_ III predicted risks (AUROC 0.824, 95 % CI 0.809–0.839), had the strongest ability to differentiate between survivors and non-survivors compared to AG (AUROC 0.660, 95 % CI 0.639–0.680), AG-corrected (AUROC 0.665, 95 % CI 0.645–0.686), SID-effective (AUROC 0.634, 95 % CI 0.613–0.655), SIG-with lactate included (AUROC 0.631, 95 % CI 0.611–0.652), SIG-without including lactate (AUROC 0.521, 95 % CI 0.500–0.542) and ‘other unmeasured ions’ (AUROC 0.679, 95 % CI 0.658–0.700) (all *p* values associated with these comparisons were < 0.01) (Table 3). Arterial lactate concentrations also had a good calibration in predicting mortality, with a relatively linear relationship to the risks of observed mortality (Appendix 2). The observed hospital mortality risk of those with an admission lactate concentration > 2 mmol/L was substantially greater than those with an admission lactate concentration ≤2 mmol/L (22.6 vs. 8.2 %, *p* = 0.001).

**Table 3:** The areas under the receiver operating characteristic curve (AUROC) of the different markers of acid-base disorders at ICU admission, with and without combining with Admission Mortality Prediction Model (MPM_0_ III) predicted risks of mortality, in differentiating between hospital survivors and non-survivors (*n* = 6878)

Acid-base markers	Mean AUROC (95 % confidence interval [CI])	
	Without MPM_0_ III	With MPM_0_ III
1. Arterial pH	0.655 (0.633–0.677)	0.805 (0.789–0.821)
2. Arterial CO_2_ tension	0.521 (0.499–0.544)	0.798 (0.782–0.814)
3. Actual bicarbonate conc.	0.676 (0.655–0.696)	0.812 (0.796–0.828)
4. Chloride conc.	0.517 (0.495–0.539)	0.801 (0.785–0.816)
5. Lactate conc.	0.701 (0.682–0.721)	0.824 (0.809–0.839)
6. Actual base excess	0.685 (0.664–0.706)	0.813 (0.797–0.829)
7. Anion gap	0.660 (0.639–0.680)	0.813 (0.798–0.828)
8. Anion gap albumin-corrected	0.665 (0.645–0.686)	0.818 (0.803–0.833)
9. Strong ion gap (SIG) with lactate	0.631 (0.611–0.652)	0.812 (0.797–0.827)
10. SIG without lactate	0.521 (0.500–0.542)	0.801 (0.786–0.817)
11. Strong ion difference-effective	0.634 (0.613–0.655)	0.809 (0.794–0.825)
12. Other unmeasured ions	0.679 (0.658–0.700)	0.820 (0.805–0.835)

The AUROC for MPM_0_ III predicted risks of mortality alone was 0.799 (95 % CI 0.783–0.814) and SAPS III predicted risks alone was 0.833 (95 % CI 0.821–0.844). The AUROC for combining lactate with anion gap albumin-corrected and MPM_0_ III, and combining lactate with SIG and MPM_0_ III were 0.830 (95 % CI 0.816–0.845) and 0.829 (95 % CI 0.815–0.844), respectively

**Fig. 2:**
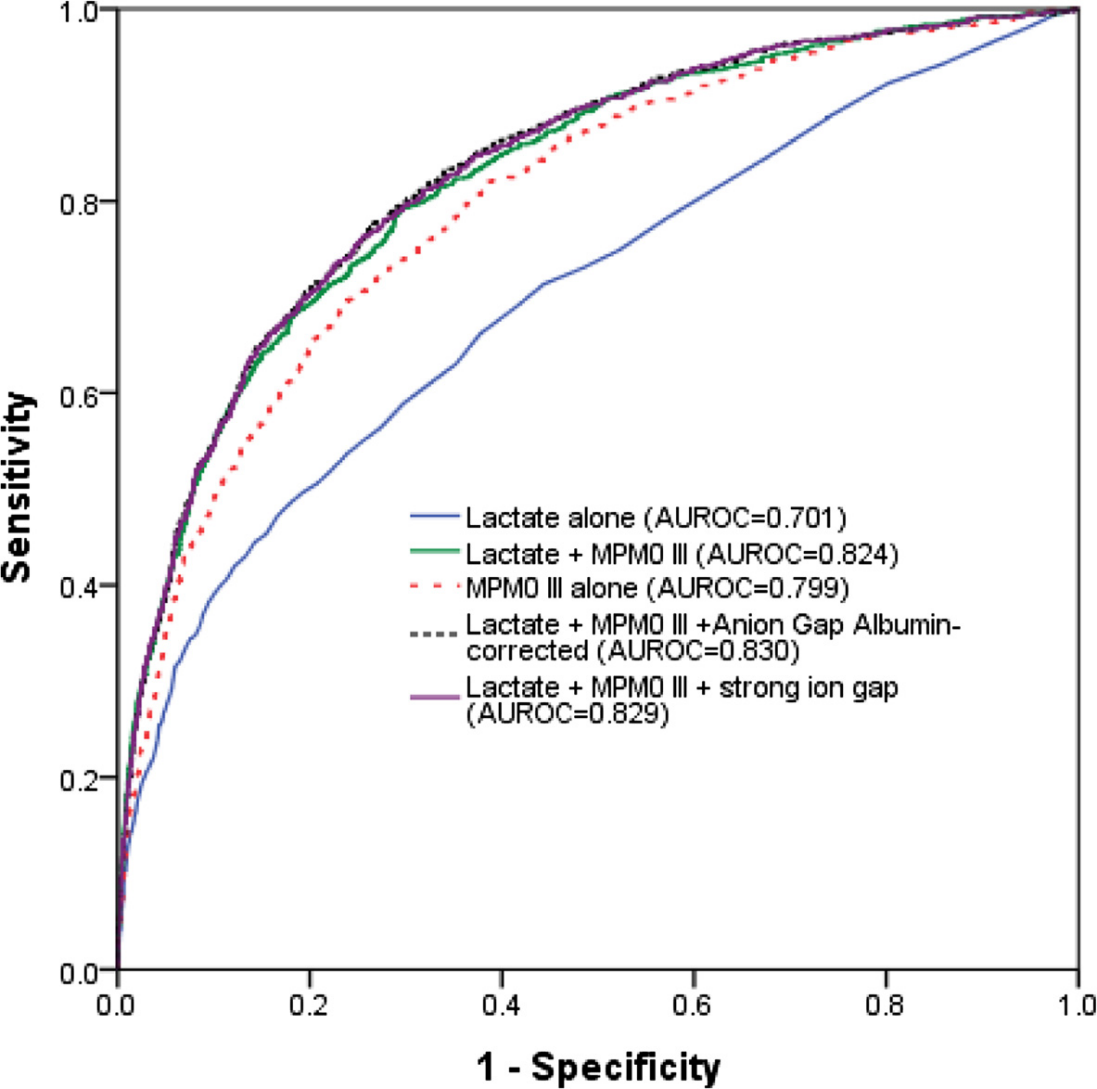
Area under the receiver operating characteristic curves (AUROC) showing improvement in discriminative ability by combining lactate with the MPM_0_ III model compared to MPM_0_ III alone, and adding anion gap albumin-corrected or strong ion gap to lactate with MPM_0_ III did not substantially further improve the latter’s ability to differentiate between survivors and non-survivors

Adding AG-corrected (AUROC 0.830, 95 % CI 0.816–0.845) or SIG-with lactate included (AUROC 0.829, 95 % CI 0.815–0.844) to a combination of lactate and the MPM_0_ III predicted risks did increase the latter’s ability to differentiate between survivors and non-survivors statistically (AUROC 0.824, 95 % CI 0.809–0.839; *p* = 0.007 and *p* = 0.014, respectively), but the magnitude of improvement was quite small (Fig. 2).

In the multivariate logistic regression model directly comparing the significance of arterial lactate with SIG-with lactate included and SIDe, arterial lactate concentrations explained about 11 % of the variability in the observed mortality and was, by far, more important than SIG-with lactate (0.6 %) and SIDe (0.9 %), while adjusting for MPM_0_ III predicted risks (65 %).

**Fig. 3:**
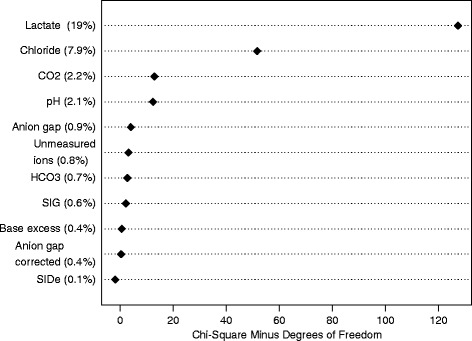
Variability in hospital mortality explained by each acid-base marker in a multivariate model including all acid-base markers and allowing each to have a U-shape relationship with mortality by a 3-knot restricted cubic spline function. *SIG* strong ion gap. *SIDe* effective strong ion difference, *CO*_*2*_ carbon dioxide, *HCO*_*3*_ actual bicarbonate

**Fig. 4:**
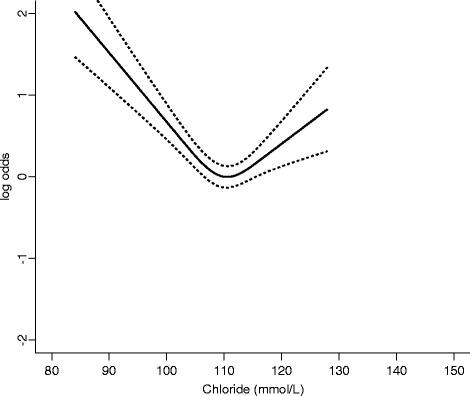
A U-shape relationship between plasma chloride concentrations and hospital mortality after adjusting for all other acid-base markers. *Dotted lines* indicate 95 % confidence interval

In the sensitivity analysis involving only patients with known cirrhosis (*n* = 167), the ability of arterial lactate (AUROC 0.734, 95 % CI 0.652–0.816) to discriminate between survivors and non-survivors remained better than SIG-with lactate (AUROC 0.644, 95 % CI 0.535–0.753), SIDe (AUROC 0.616, 95 % CI 0.518–0.714) or even bilirubin concentration (AUROC 0.604, 95 % CI 0.500–0.707). Similarly, combining arterial lactate concentrations with SAPS III predicted risks of mortality (AUROC 0.852) was still better than the combination of SIG-with lactate (AUROC 0.836) or SIDe (AUROC 0.835) with the SAPS III predicted risks in differentiating between survivors and non-survivors. After centring the U-shape predictors before the ROC analyses, the improvements in AUROC for pH, chloride, bicarbonate and arterial carbon dioxide tension were small (all increments < 0.015) and remained inferior to using lactate alone. When all acid-base markers were analysed simultaneously in a multivariate model with a 3-knot restricted cubic spline function allowing non-linearity for all predictors in the model, lactate explained 19 % of the variability in hospital mortality and remained as the strongest predictor for hospital mortality compared to other acid-base markers (Fig. 3). In this direct comparison of all acid-base markers while allowing predictors to assume a U-shape relationship, chloride became the second most important acid-base predictor (Fig. 4).

## Discussion

This study showed that many markers of acid-base status of critically ill patients on ICU admission were significantly different between survivors and non-survivors. Of all the acid-base markers assessed, arterial lactate concentration had the best discrimination and was better than SIG (with and without including lactate as part of its calculations) in discriminating between survivors and non-survivors—both when it was analysed on its own and simultaneously with the MPM_0_ III predicted risks of mortality. Adding SIG to a combination of lactate and MPM_0_ III predicted risks also did not greatly improve our ability to predict mortality of critically ill patients. These findings are clinically relevant and require further discussion.

Evidence suggests that Stewart’s approach to acid-base may help us to identify important metabolic acid-base abnormalities that are not apparent by using the physiological or BE approach alone [18]. There were also studies showing that SIG or SIDe could be better than BE, AG or lactate in predicting outcomes of critically ill patients [5, 6, 19–22]. However, many of these studies are relatively small, with the two largest studies (involving 410 and 935 patients) both showing an insignificant advantage by using SIG and SIDe instead of standard markers of acid-base disturbances in predicting mortality of critically ill patients [3, 23, 24]. To the best of our knowledge, this is the largest study (*n* = 6878) assessing the prognostic significance of SIG (with and without including lactate) relative to 10 other acid-base markers in a heterogeneous group of critically ill patients, and our findings are consistent with the data from two largest published studies [3, 23]. Indeed, both these studies showed that lactate had the strongest ability to differentiate between survivors and non-survivors compared to SIG or SIDe (AUROC lactate: 0.67 and 0.77 vs. AUROC SIG: 0.62 and 0.67, respectively), suggesting that arterial lactate concentration should always be considered [1], regardless of the approach used to assess acid-base disturbances in the critically ill. This result also supports the hypothesis that lactate concentration is a preferred resuscitation target in patients with critical illness [25–27].

So, why was SIG or SIDe not better than arterial lactate in predicting mortality in our patients? First of all, it may be too simplistic to assume, by intuition, that the prognostic significance of SIG with lactate should be better than using lactate alone just because the former includes lactate and also other biochemical variables. Although the SIG has the ability to reflect acid-base abnormalities as a result of different pathologies, its prognostic significance can also be confounded by changes in the underlying elements of SIG in different directions. Lactate is an anion and an elevated lactate concentration would be, at least in part, accounted for by an abnormal SIG, or SIDe. However, hyperlactataemia can also be ‘concealed’ with a relatively normal SIG, bicarbonate concentration, BE or SIDe, due to coexisting hypochloraemic alkalosis [28]. As such, by combining not so important predictors with an important predictor of mortality (e.g. lactate) within the calculation of SIG, it has the potential to reduce the prognostic significance of SIG. Furthermore, an increase in other measured and unmeasured anions including ketoacids, formate, oxalate, salicylate, sulphate and phosphate [1], leading to an increase in SIG (and a decrease in SIDe) without hyperlactataemia, also does not have the same prognostic significance as lactic acidosis and is more amendable to specific supportive therapy that can directly improve patient outcomes.

Second, acute and chronic and liver diseases were uncommon in our patients (2 %). Previous studies have showed that patients with liver diseases often have accumulation of unmeasured anions in addition to lactate [15, 16]; hence, SIG and SIDe may have a stronger prognostic significance than using lactate alone for these patients. Third, we assessed the prognostic significance of all the markers of acid-base disturbances in blood samples obtained within the first hour of ICU admission. It is well established that administering large quantity of intravenous fluid to critically ill patients can alter their acid-base status through multiple mechanisms, including inducing hyperchloraemic acidosis and dilutional hypoalbuminaemia. Whether SIG and SIDe may have a stronger association with mortality than arterial lactate in the later phase of critical illness after a large quantity of intravenous fluid is used for resuscitation remains uncertain, and this merits further evaluation.

Although ‘other unmeasured ions’ (or BE gap) is theoretically similar to SIG calculated without lactate and has received considerable attention over the years [29, 30], it is not simple to use and was not as good as lactate, both alone and in combination with MPM_0_ III predicted risks, in predicting mortality of the critically ill. In addition, our study also showed that arterial carbon dioxide tension (AUROC 0.521) and chloride concentrations (AUROC 0.517) were not as important as arterial lactate concentrations in discriminating between survivors and non-survivors. This may be due to the fact that the underlying causes for both respiratory acidosis and hyperchloraemia are usually obvious to the treating clinicians and are also more readily treatable than lactic acidosis using mechanical ventilation and sodium bicarbonate or diuretics, respectively.

This study has some limitations. First, although we had included a large number of patients and our results were consistent with largest published studies [3, 23], this was still a single-centre study potentially limiting its general applicability and to different specific subgroups of critically ill patients, especially those with acute liver failure. Second, inherent to all diagnostic tests, noting abnormal results from a diagnostic test is not necessarily translatable to improved outcomes, unless the underlying pathological process reflected by the diagnostic test can be identified and modified. Finally, temporal changes in acid-base markers during the course of critical illness and after different interventions are common and may affect the prognostic significance of each acid-base marker differently. Whether serial lactate concentrations are the most important acid-base marker to be targeted in the critically ill remains uncertain [25, 27], and this merits further investigation.

## Conclusions

In conclusion, many markers of acid-base status in critically ill patients were significantly different between survivors and non-survivors. The prognostic significance of SIG was modest and inferior to arterial lactate concentration for the critically ill. Of all the acid-base markers assessed in this large cohort study, arterial lactate concentration had the best discrimination—both when it was analysed on its own and simultaneously with the MPM_0_ III predicted risks of mortality or other acid-base markers while allowing a U-shape relationship for all acid-base markers—suggesting that arterial lactate concentration is the important marker of acid-base disorders in determining mortality outcome of critically ill patients. Lactate concentration should always be considered regardless of which approach is used to interpret acid-base disturbances in critically ill patients; a high lactate concentration can be considered as a simple, and yet, important warning acid-base marker for patients who are at risk of dying from critical illness.

## Figures and Tables

**Fig. 5:**
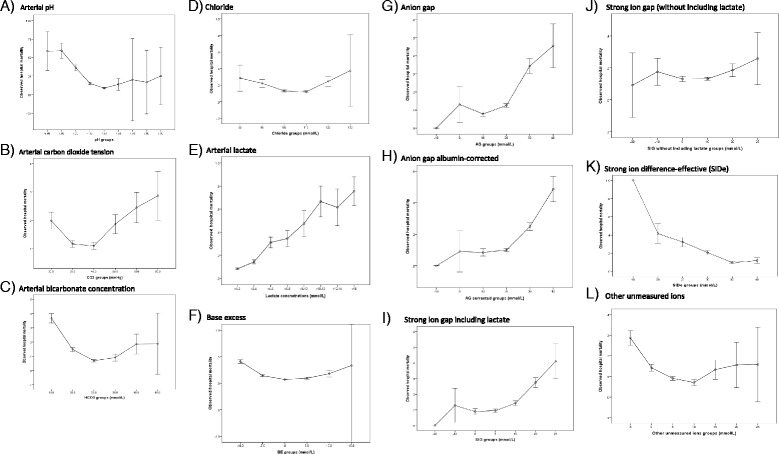
**a** Arterial pH. **b** Arterial carbon dioxide tension. **c** Arterial bicarbonate concentration. **d** Chloride. **e** Arterial lactate. **f** Base excess. **g** Anion gap. **h** Anion gap albumin-corrected. **i** Strong ion gap including lactate. **j** Strong ion gap (without including lactate). **k** Strong ion difference (SIDe). **l** Other unmeasured ions

## Appendices

### Appendix 1

Calculated acid-base variables:

Anion gap = [Na^+^] + [K^+^] − [Cl^−^] − [HCO_3_^−^]

Anion gap albumin-corrected = anion gap + 0.25 × (42 − observed albumin in g/L)

Strong ion difference-effective (SIDe) = [HCO_3_^−^] + [albumin] × (0.123 × pH − 0.631) + 2 × [phosphate] × (0.309 × pH − 0.469)

Strong ion difference-apparent (SIDa) = [Na^+^] + [K^+^] + 2 × [Ca^2+^] + 2 × [Mg^2+^] − [Cl^−^] − [lactate]

Strong ion difference-apparent (SIDa with lactate included) = [Na+] + [K+] + 2 × [Ca^2+^] + 2 × [Mg^2+^] − [Cl^−^]

Strong ion gap = (strong ion difference-apparent) − (strong ion difference-effective)

Strong ion gap with lactate included = (strong ion difference-apparent with lactate) − (strong ion difference-effective)

‘Other unmeasured ions’ = base excess (BE) − ‘protein effect’ − ‘chloride effect’ − ‘water effect’ = BE − (0.148 × pH − 0.818) × (42 − measured albumin in g/L) − (102 − measured [Cl^−^] × 140/measured [Na^+^]) − 0.3 × (measured [Na^+^] − 140)

Arterial blood gases including lactate, chloride concentrations were measured by ABL800 FLEX blood gas analyser (Radiometer®, Copenhagen, Denmark) (bias and precision are described in http://www.radiometeramerica.com/~/media/files/radiometercomcloneset/rame/manuals/abl800/989-963i%20abl800%20reference%20manual%20-%20english%20us.Pdf?bcsi_scan_313cddce030931be=sI53xPnlknYjOddC3jutx1ef6TcKAAAA7PkeTA==&bcsi_scan_filename=989-963i%20abl800%20reference%20manual%20-%20english%20us.Pdf ).

All concentration measurements used in the calculations are based on millimole per litre.

### Appendix 2

Scatter plots showed no extreme U-shape relationships between observed risks of mortality and different markers of acid-base status which may substantially compromise the receiver operating characteristic (ROC) analyses. All error bars in the graphs in Appendix 2 signify 95 % confidence interval of the observed hospital mortality risk for subgroup of patients with the value of the associated acid-base marker on admission to the intensive care unit (Fig. 5).

## Abbreviations

AG: Anion gap
APACHE: Acute Physiology and Chronic Health Evaluation
AUROC: Area under the receiver operating characteristic curve
ICU: Intensive care unit
MPM: Mortality Prediction Model
SAPS: Simplified Acute Physiology Score
SID: Strong ion difference
SIG: Strong ion gap
